# An aptamer-based shear horizontal surface acoustic wave biosensor with a CVD-grown single-layered graphene film for high-sensitivity detection of a label-free endotoxin

**DOI:** 10.1038/s41378-019-0118-6

**Published:** 2020-02-10

**Authors:** Junwang Ji, Yiquan Pang, Dongxiao Li, Zheng Huang, Zuwei Zhang, Ning Xue, Yi Xu, Xiaojing Mu

**Affiliations:** 1 Key Laboratory of Optoelectronic Technology & Systems, Ministry of Education, International R & D Center of Micro-nano Systems and New Materials Technology, Chongqing University, 400044 Chongqing, China; 2 School of Chemistry and Chemical Engineering, Chongqing University, 400030 Chongqing, China; 3 Department of Applied Physics, Chongqing University, 401331 Chongqing, China; 4 Chongqing Acoustic-Optic-Electric Corporation, China Electronic Technology Group Corporation, 400060 Chongqing, China; 5 Institute of Electronics, Chinese Academy of Sciences, 100190 Beijing, China

**Keywords:** Electronic properties and materials, Nanobiotechnology, Electronic properties and materials, Nanobiotechnology

## Abstract

The thickness of the sensitive layer has an important influence on the sensitivity of a shear horizontal surface acoustic wave (SH-SAW) biosensor with a delay-line structure and lower layer numbers of graphene produce better sensitivity for biological detection. Therefore, a label-free and highly sensitive SH-SAW biosensor with chemical vapor deposition (CVD-)-grown single-layered graphene (SLG) for endotoxin detection was developed in this study. With this methodology, SH-SAW biosensors were fabricated on a 36° Y-90° X quartz substrate with a base frequency of 246.2 MHz, and an effective detection cell was fabricated using acrylic material. To increase the surface hydrophilicity, chitosan was applied to the surface of the SLG film. Additionally, the aptamer was immobilized on the surface of the SLG film by cross-linking with glutaraldehyde. Finally, the sensitivity was verified by endotoxin detection with a linear detection ranging from 0 to 100 ng/mL, and the detection limit (LOD) was as low as 3.53 ng/mL. In addition, the stability of this type of SH-SAW biosensor from the air phase to the liquid phase proved to be excellent and the specificity was tested and verified by detecting the endotoxin obtained from Escherichia coli (E. coli), the endotoxin obtained from Pseudomonas aeruginosa (P. aeruginosa), and aflatoxin. Therefore, this type of SH-SAW biosensor with a CVD-grown SLG film may offer a promising approach to endotoxin detection, and it may have great potential in clinical applications.

## Sensors: High-sensitivity biosensor based on single-layered grapheme film

Researchers in China have invented a biosensor which can detect molecules from bacterial cell walls at just a few nanograms per milliliter. The device is a shear horizontal surface acoustic wave (SH-SAW) biosensor. SH-SAW devices are cheap, sensitive, and can be used label-free, but their performance depends on the characteristics of the sensitive layers. A team led by Xiaojing Mu of Chongqing University used chemical vapor deposition to engineer a device with a single-layered grapheme film as its sensitive layer. They tested its sensitivity in detecting endotoxins, toxic molecules in the cell walls of some bacteria, and found that it could detect quantities as low as 3.53 ng/mL. Further testing showed that the device did not detect glucose or aflatoxin, demonstrating its specificity. Devices based on this design could be useful in clinical settings.

## Introduction

Endotoxins are complex lipopolysaccharides (LPS) that form the cell walls of various gram-negative bacteria. Structurally, LPS consists of lipid A, core polysaccharide, and O-polysaccharide side chains. Among them, lipid A is the main component of the bacterial endotoxin, which determines its toxicity, the O-polysaccharide side chain is highly variable among different bacteria, and the specificity determines the serotype of bacteria. Endotoxins are responsible for the toxic effects that cause fevers,^1^ septic shock,^2^ and sepsis.^3,4^ Biosensor based endotoxin detection has been widely investigated recently,^5,6^ and many methods have been investigated to identify endotoxins: hydrophobic interactions,^7^ localized surface plasmon resonance (LSPR),^8^ mass spectrometry,^9^ optical methods,^10^ the electronic tongue,^11^ the voltammetric method,^12^ and electrochemistry.^13–17^ However, these methods have some disadvantages, such as high costs, a long processing time and the requirement of labeled markers. Thus, the demand for rapid, simple operations and low costs is increasing.

Shear horizontal surface acoustic wave (SH-SAW) biosensors have been widely reported owing to aspects such as their high selectivity and/or sensitive detection of deoxyribonucleic acid (DNA),^18^ proteins,^19^ and cells.^20–23^ SH-SAW biosensors have the advantages of low cost, operational simplicity, and high sensitivity, and these biosensors can be used in label-free and real-time monitoring. Additionally, SH-SAW biosensors are especially suitable for biological detection in a liquid phase, as the particle displacement is parallel to the SAW propagation direction. Nanogold film have traditionally been used as sensitive layers for these biosensors. In contrast to these materials, the thickness of nanogold films is usually in the range of tens of nanometers, which would undoubtedly affect the performance of a biosensor.

Compared to noble (Au)-based electrode materials, carbon-based materials show some advantages. That is, the presence of the sp^2^ hybridized carbon atom center in their structural backbone provides a route for surface modification. Sensing devices produced with bare-carbon-based materials were able to determine analytes down to trace levels. Additionally, its use as the sensing material could enhance the signal in biosensors. Moreover, graphene is exceptionally biocompatible if used as a sensitive layer for the immobilization of biomolecules.^24^ Therefore, graphene-related materials have recently attracted great interest from researchers in the biosensor field.^25^ Among them, graphene oxide (GO) is widely applied as the sensitive layer of the biosensor.^26–31^ However, in the case of GO, the sensitivity strongly depends on the lateral size, the layer number, and the number of oxygen-containing groups.^32^

The rapid development of the chemical vapor deposition (CVD) technique in recent years has enabled the production of large-area, high-quality graphene films with good structural controllability, which is particularly beneficial for the fabrication of sensing devices.^33^ Biosensors fabricated with graphene have been widely investigated for the detection of viruses,^34^ bacteria,^35^ proteins,^36^ and nucleic acids.^37^ In addition, SLG-based biosensors have demonstrated excellent biocompatibility, conductivity and high sensitivity.^38,39^ Recently, researchers have used a finite element analysis (FET) DNA biosensor with SLG to reach a detection limit of 10 fM for target DNA with a dynamic range of 10 fM to 100 pM,^40^ indicating that lower layer numbers of graphene produce better sensitivity for the biosensor. A Hall effect biosensor with ultraclean gold-transferred SLG for the detection of DNA hybridization could increase the concentrations of target or one-base mismatched DNA from 1 pM to 100 nM.^41^ Thus far, SAW-based biosensors for the detection of an endotoxin with SLG have not yet been reported.

In this work, we present a highly sensitive and specific SH-SAW biosensor with SLG film in the sensitive area; the SLG was transferred onto a 36° Y-90° X quartz substrate using polymethyl methacrylate (PMMA) after CVD growth. In addition, the aptamer was immobilized on the sensitive area by glutaraldehyde (GA) cross-linked chitosan (CS).^42–44^ The LPS applied in this study was obtained from E. coli 055:B5 (L4524), which was extracted by benzene. Finally, the performance of the biosensor was proven to linearly detect an endotoxin in a wide range from 0 to 100 ng/mL, and the biosensor exhibited a detection limit of 3.53 ng/mL. In contrast, a recent report^45^ on a fluorescent aptamer-based probe for the determination of the LPS of Gram-negative bacteria exhibited a detection limit of 8.7 ng/mL. In addition, the selectivity was verified by distinguishing the endotoxin from endotoxin obtained from P. aeruginosa and aflatoxin, and the stability proved to be excellent. Overall, this type of detection strategy may have great potential in future applications.

## Materials and methods

### Materials and reagents

The copper foils (purity: 99.8%) were purchased from Shenzhen Changda Sheng Electronics Co., Ltd. The PMMA was obtained from Wenzhou Yuanteng Plastic Co., Ltd. The 36° Y-90° X quartz substrates with Au (purity: 99.9999%) were obtained from Wuxi Haoda Electronics Co., Ltd. The hydrochloric acid, acetone, CS, GA and etching solution (FeCl_3_/HCl) were purchased from Chongqing Xingguanghuabo Company. The aptamer (NH_2_-5′-CTT CTG CCC GCC TCC TTC C- TAG CCG GAT CGC GCT GGC CAG ATG ATA TAA AGG GTC AGC CCC CCA -GGA GAC GAG ATA GGC GGA CAC T-3′) was synthesized by Bioengineering (Shanghai) Co., Ltd.

### Fabrication of the SH-SAW biosensor

The SH-SAW biosensor with a delay-line structure was fabricated with a typical MEMS process,^46^ including lithography development, vacuum magnetron sputtering, and lift-off on a 36° Y-90° X quartz substrate with a thickness of 0.5 mm and a diameter of four inches. A Cr/Au film (40/100 nm) was sputtered over the entire wafer surface by magnetron sputtering, and then the IDTs and electrodes were formed by immersing the wafer into acetone to lift off the excess metal. To increase the adhesion of Au, Cr was used as an adhesion layer.

### The growth and transfer of high-quality single-layered graphene

The SLG film was synthesized with CVD on copper foil. Prior to growth, the copper foil was thoroughly cleaned with hydrochloric acid and cut into a size of 6 mm × 3 mm. The copper foil was placed in a tube furnace and the gas path was kept sealed. The quartz tube was vacuumed and then hydrogen gas was passed into the tube to keep it at atmospheric pressure. The air in the airway was exhausted three times to keep the vacuum pump working continuously with constant pressure (100 Pa). The system was heated to 1050 °C in 100 min with a flow of H_2_ (20 sccm), and then kept under those conditions for 10 min. Next, a flow of CH_4_ (35 sccm) was introduced into the system, which was kept under this condition for 20 min, and graphene was grown on the surface of the copper foil. Finally, the chamber was naturally cooled down to room temperature under an H_2_/Ar atmosphere, and then the gas was closed off.

### Transfer of high-quality single-layered graphene using PMMA

**Fig. 1:**
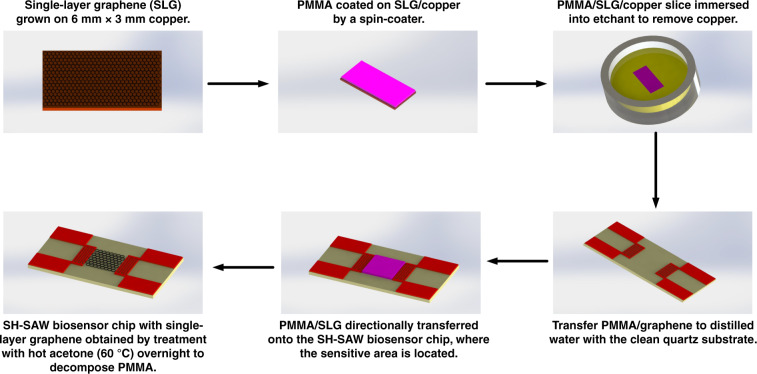
Transfer process of CVD-grown single-layered graphene using a PMMA supporting layer.

As shown in Fig. 1, prior to the transfer of graphene on the quartz substrate, one side of the copper foil was treated with plasma to clean up the graphene. This action was performed to increase the ease of corrupting the copper foil in the later stage. Next, a layer of polymethyl methacrylate (PMMA) was spin-coated on the other side of the copper foil. After the PMMA layer was baked, the copper foil was placed on the surface of the FeCl_3_/HCl etching solution. Half an hour later, the graphene/PMMA was transferred to distilled water for two hours to clean the surface impurities when the copper foil was corroded. After rinsing with deionized water, a clean glass plate was used to support the PMMA/graphene layer (6 mm × 3 mm in area). After baking at 60 °C for 2 h, the sample was immersed in hot acetone (60 °C) for 10 min to dissolve the PMMA followed by annealing in the CVD furnace under a H_2_/Ar (20/80 sccm) atmosphere at 450 °C for 2 h to remove the rest of the PMMA. High-quality single-layered graphene was then obtained. Finally, for the sensitive layer, the single-layered graphene was transferred onto the sensitive area of the 36° X-90° Y quartz substrate to characterize the performance of the biosensor.

### Related theory

In this study, a delay line structure with a single channel was designed, which generally consists of input and output IDTs that were mounted on the 36° Y-90° X quartz substrate. The SH-SAW is simulated by the input IDTs, and then propagates along the surface of the sensitive area through the delay line which lies between the input IDTs and the output IDTs. The SH-SAW resonant frequency can be calculated by the following equation:

1 $$f_0 = V_{\mathrm{s}}/2(a + b),$$

where *f*_0_ is the resonant frequency of the device (MHz), *V*_s_ is the SH-SAW propagating velocity on the piezoelectric substrate (m/s), *a* is the width of the IDT fingers and *b* is the gap between the IDT fingers (μm). Generally, a simple situation, namely, $\lambda = a = b$ is adopted; thus, *f*_0_ can be further expressed as

2 $$f_0 = V_s/4\lambda$$

The amplitude and the phase velocity varied with the change in the mass loading on the propagation path. The phase change was collected by the detection system. The phase change and the amplitude change are expressed as follows

3 $$\Delta {\mathrm{Ph}} = 360fl\left(\frac{1}{{V_0}} - \frac{1}{{V_1}}\right),$$

4 $$\Delta {\mathrm{Amp}} = fl\left(\frac{{a_1}}{{V_1}} - \frac{{a_0}}{{V_0}}\right),$$

where $\Delta {\mathrm{Ph}}$ is the phase shift of the SH-SAW biosensor (°), *f* is the frequency (Hz), *l* is the propagation length (m), *V*_0_ is the velocity before loading (m/s), *V*_1_ is the velocity after loading (m/s), $\Delta {\mathrm{Amp}}$ is the amplitude change of the SH-SAW biosensor (d*B*), *a*_0_ is the propagation loss before loading (d*B*/*λ*), and *a*_1_ is the propagation loss after loading (d*B*/*λ*).

### Immobilization of the aptamer on single-layered graphene

Prior to the immobilization of the aptamer, the CS solution (0.2%) was dropped on the surface of the sensitive area and dried at room temperature to increase the hydrophilicity of the SLG film. Next, 5 μL of GA solution in acetic acid at 2.5% (m/m) (50 mmol/L, PH = 7.4) was dropped onto the surface of the modified sensitive area, and then the sensitive area was soaked in a GA solution for 2 h to link the amino-groups in the CS. After that, the SH-SAW biosensor was rinsed with deionized water to clean the residual reagent, and then dried at room temperature. The aptamer solution (10 nmol/L) was then pumped into the reaction cell, so that the aptamer could be chemically bonded to the aldehyde-groups in the GA. The sensitive area was then washed with deionized water to remove the unreacted aptamer. After that, the endotoxin solution (0–100 ng/mL) was pumped into the reaction cell, and then the aptamer would specifically bind to the endotoxin. Therefore, the endotoxin could be detected by an SH-SAW biosensor with a CVD-grown SLG.

### Detection procedures

Prior to the experiment, a homemade detection cell was fabricated to increase the ease of operation (Fig. S1). SMA cables were used to connect the vertical network analyzer (Agilent, E5080A) (Fig. S1D), and the phase signals were monitored and recorded by a biosensor monitoring system based on the LabVIEW software. First, the surface of the sensitive area was treated with CS and GA to ensure the linking of the related groups, and then the detection biosensor chip was put into the groove of the detection cell. The phase (*P*_0_) of the SH-SAW biosensor was then monitored until a steady baseline was observed in the liquid phase environment. Next, the aptamer solution was pumped into the reaction cell to link the aldehyde in GA until the phase reached a stable value, and the steady-state phase was taken as *P*_1_. Finally, the endotoxin solution was pumped into the reaction cell, and the biosensor was then maintained at 37 °C for the reaction. When the resonance phase reached a stable value, the steady-state phase was taken as *P*_2_. The phase shift attributed to the aptamer-endotoxin reaction was calculated by the equation: $\Delta P = P_2 - P_1$.

### Selectivity assessment

To assess the selectivity, SH-SAW biosensor chips were employed to detect aflatoxin and the endotoxin obtained from Pseudomonas aeruginosa (P. aeruginosa). The aptamer was immobilized on the sensitive surface of the SH-SAW biosensor chip, and then the aflatoxin and the endotoxin obtained from P. aeruginosa were injected into the reaction cells to verify the selectivity.

### Electrical measurements and characterizations

Detailed information regarding the electrical measurements and the characterizations of the equipment used in this study are given in SI-1.2.

## Results and discussion

### The principles and the structure of the SH-SAW biosensor with SLG

**Fig. 2:**
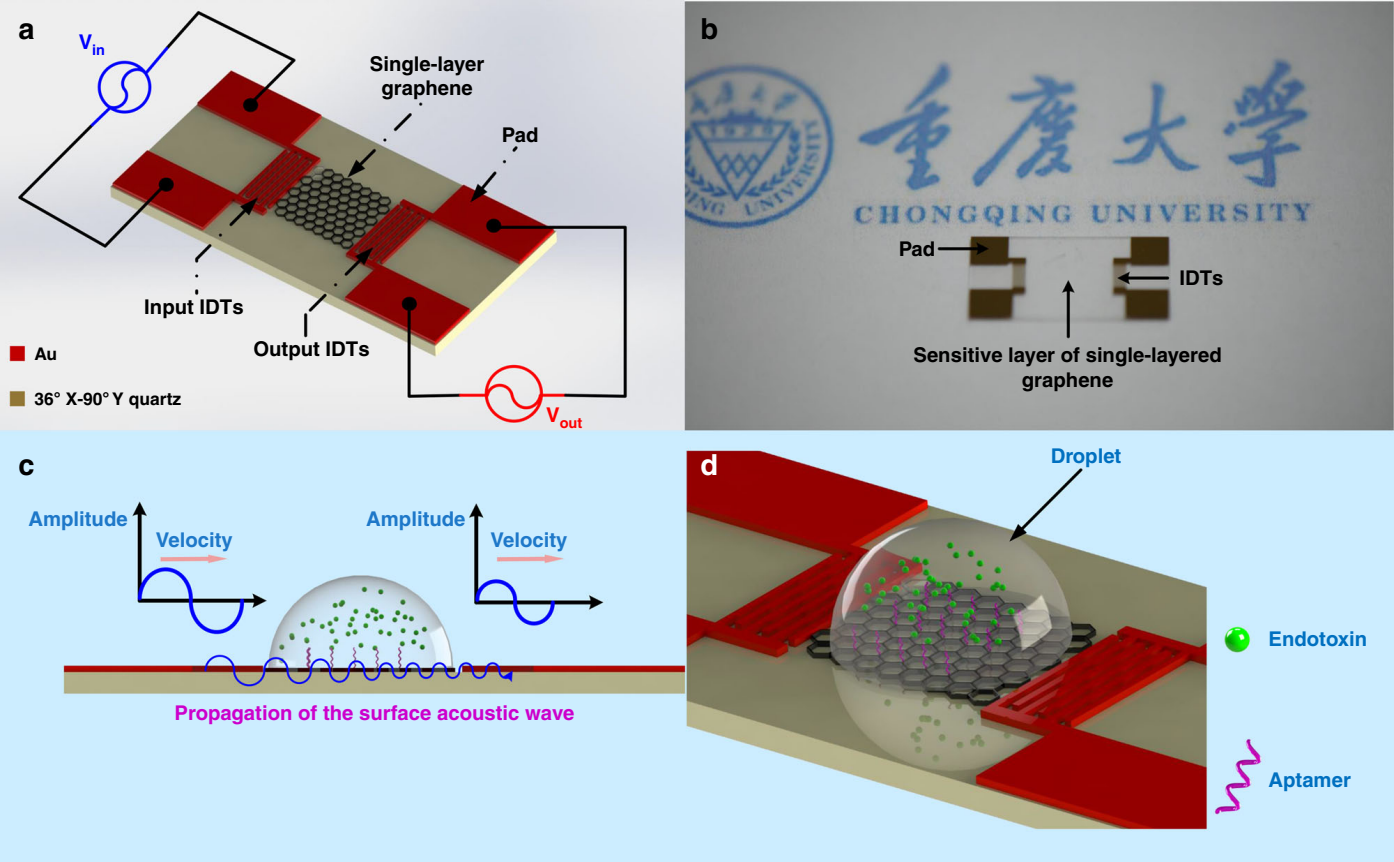
The principles and detailed configuration of the SH-SAW device with SLG. **a** Schematic of the SH-SAW biosensor chip and the working principles. **b** Photograph of the fabricated SH-SAW biosensor chip. **c**, **d** Principles and schematic illustration of endotoxin detection on the SLG surface.

The detailed configuration of the SH-SAW device with SLG is shown in Fig. 2. The SLG was deposited on the sensitive area of the SH-SAW device as the sensitive layer (Fig. 2a). Additionally, the real product of the SH-SAW biosensor with the SLG is shown in Fig. 2b. The specific parameters of the SH-SAW device are shown in SI-2.1. To illustrate this approach for the high sensitivity detection of the endotoxin, the principle of the SH-SAW biosensor is demonstrated in Fig. 2c, d. The aptamer was first immobilized on the SLG film, and then the endotoxin was captured based on the specific interaction between the aptamer and the endotoxin. The phase shift of the SH-SAW biosensor was thus observed for the mass-change in the sensitive area. Additionally, the propagation characteristics of the SH-SAW is verified by the COMSOL 5.2a software (Fig. S3).

**Fig. 3:**
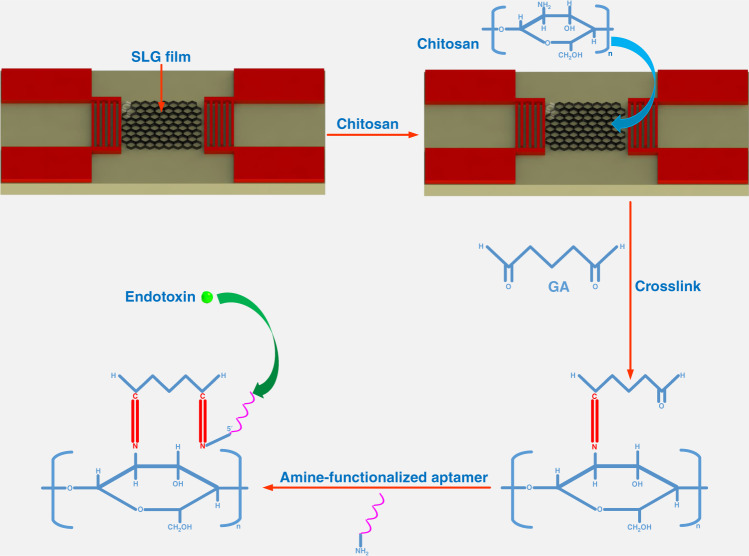
The surface functionalization process including the surface modification with chitosan and the glutaraldehyde to amine-functionalized aptamer immobilization processes.

The aptamer immobilization processes are shown in Fig. 3. The CS was first immobilized on the SLG film, and then the amino groups in the CS reacted with the aldehyde in GA to form C=N bonds. After that, the aldehydes groups in GA reacted with the amine-functionalized aptamer. It was then ready for the specific detection of an endotoxin.

### Characterizations of SLG

**Fig. 4:**
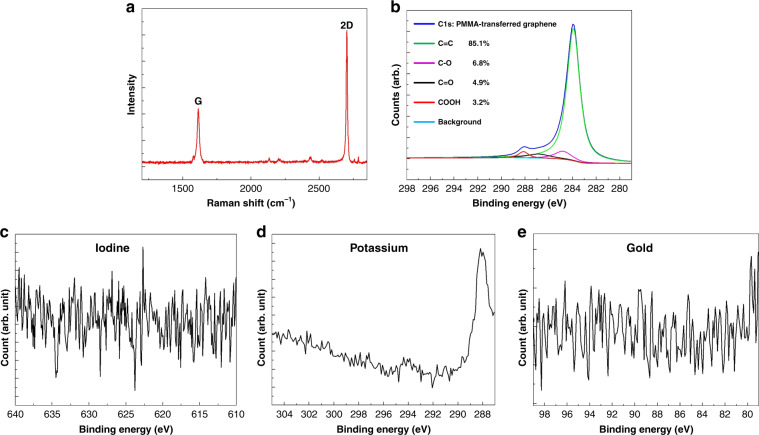
The characterization results of the SLG. **a** Raman spectra of CVD-grown SLG. **b** XPS spectra of C1s and **c**–**e** other elements of the SLG film.

The Raman spectra of the SLG film are presented in Fig. 4a. The Raman spectra exhibit the characteristic peak of a high-quality SLG: a sharp G-band (≈1600 cm^−1^) and a sharp 2D-band (2699–2720 cm^−1^).^47^ The *I*_2D_/*I*_G_ ratio (2.37) shows the excellent quality of the SLG.

To examine and analyze the surface chemical compositions of the SLG, X-ray photoelectron spectroscopy (XPS) was conducted. As shown in Fig. 4b, the blue line represents the raw spectrum. To fit the C1s spectrum, four components were obtained: the sp^2^ component of C=C (green line, at 284.6 eV) and the oxygen-containing groups including C–O (purple line, at 286.1 eV), C=O (black line, at 287.1 eV), and COOH (red line, at 288.7 eV).^48^ The contents of the chemical states of these elements at different peak positions were calculated using the peak area. As shown in Fig. 4c–e, the data indicated that impurities could not be found on the surface of the SLG film. Therefore, the PMMA was completely removed from the graphene surface due to hot acetone.

**Fig. 5:**
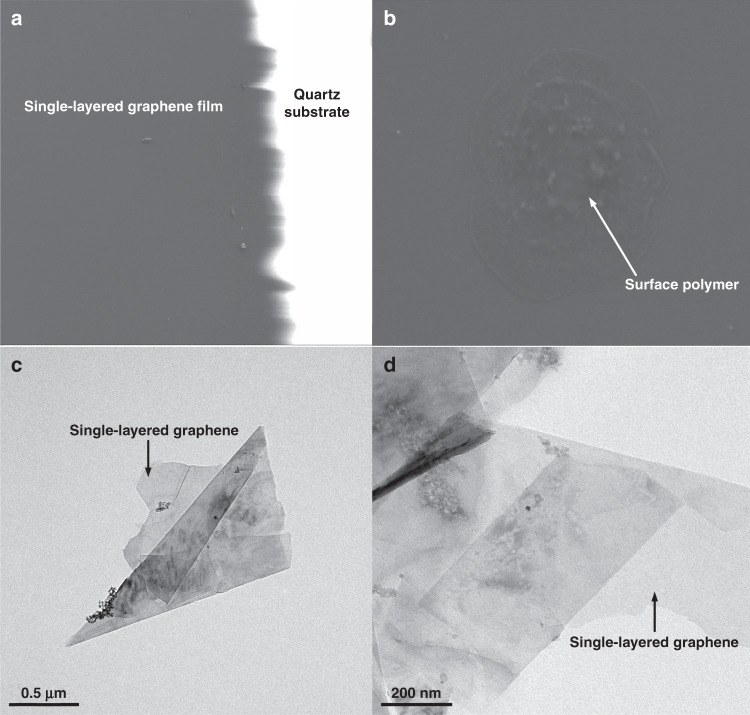
The SEM images of CVD-grown single-layered graphene film in the sensitive area. **a**, **b** SEM images of CVD-grown single-layered graphene film in the sensitive area of the SH-SAW biosensor chip. **c**, **d** TEM images of single-layered graphene obtained from the sensitive area of the SH-SAW biosensor chip.

Additionally, scanning electron microscopy (SEM) and transmission electron microscopy (TEM) were used to characterize the surface topography of the sensitive area and the single-layered graphene obtained from the sensitive area respectively. Figure 4a shows that the black area is the single-layered graphene film grown in the sensitive area by CVD, and the white area is the quartz substrate. It is known that graphene possesses good electrical conductivity; hence, this obvious dividing line (Fig. 5a) indicates that the single-layered graphene kept a certain distance from the IDTs. Therefore, short circuit of the IDTs could be avoided. Figure 5b shows polymers on the surface of the single-layered film. As shown in Fig. 5c, d, the morphology of the single-layered graphene is very clear. To prepare test samples, graphene was obtained from the sensitive area. Next, the samples were made into a suspension by ultrasonic oscillation in alcohol. Finally, the suspension was dropped on ultrathin carbon mesh for testing with TEM.

### Characterization by AFM

**Fig. 6:**
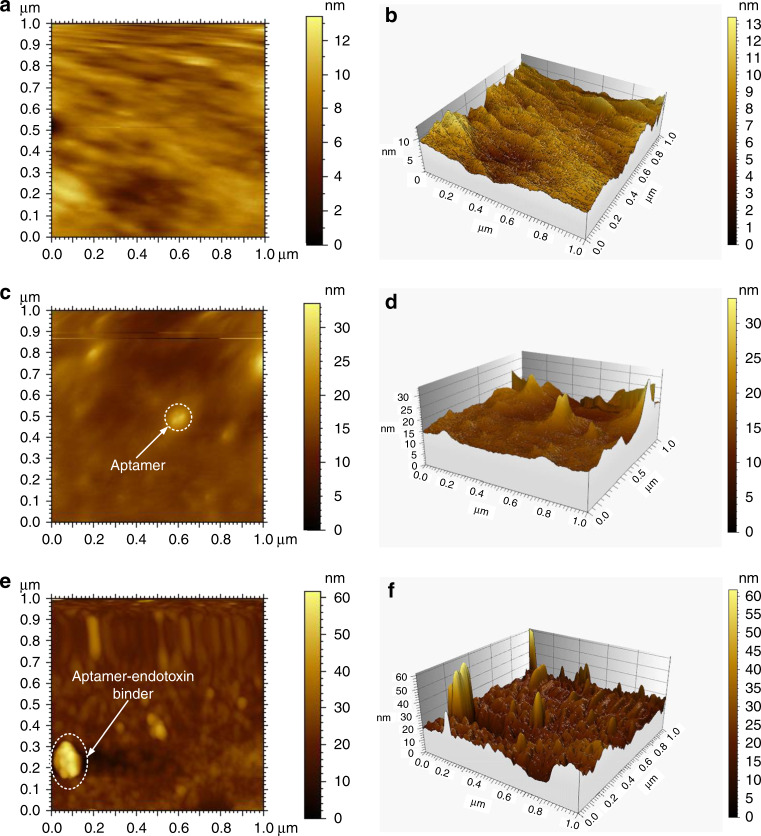
The AFM images the surface morphology of the sensitive area with single-layered graphene during the experiments. **a**, **b** AFM images of the surface morphology of the sensitive area with single-layered graphene. **c**, **d** AFM images of the surface morphology of the sensitive area after the aptamer was immobilized. **e**, **f** AFM images of the surface morphology of the sensitive area after the specific binding of the aptamer and the endotoxin.

The AFM images are presented in Fig. 6. The surface morphology of the sensitive area with single-layered graphene is illustrated in Fig. 6a, b, which show that the surface morphology is very smooth. Figure 6c, d indicate that the surface morphology was remarkably changed after the aptamer was immobilized in the sensitive area. After the endotoxin was pumped into the reaction cell, the obvious change in the surface morphology (Fig. 6e, f) indicated that the specific binding of aptamer and endotoxin occurred in the sensitive area. The parameters used for the AFM technique in the morphological analysis were the following: imaging resolution was 256, the scanning speed was 0.7 Hz, the imaging mode was the tapping mode, and the imaging force setpoint was as follows: 2.9313 P: 1.4 I: 0.7.

### The analytical performance of the SH-SAW biosensor with SLG

**Fig. 7:**
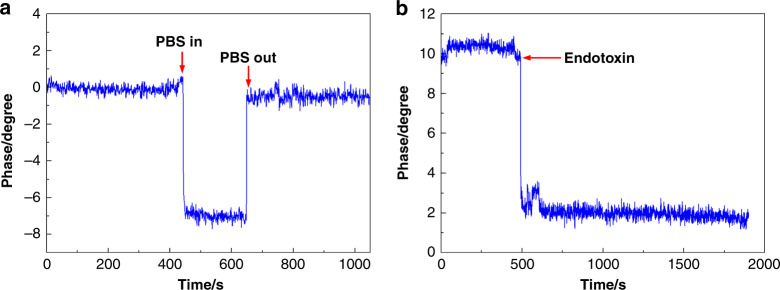
The stability of the SH-SAW biosenor and the verification of aptamer role. **a** Phase changes of the SH-SAW biosensor chip in liquid and air, and **b** phase shift induced by pumping the endotoxin into the sensitive area without the aptamer immobilized in it.

Stability is an important factor used to evaluate the performance of an SH-SAW biosensor. Initially, the PBS solution was pumped into the reaction cell while the phase signal was kept stable in the air phase. Then the PBS solution was pumped out of the reaction cell. The result (Fig. 7a) revealed that the phase signal could remain in the stable state when sudden environmental changes occurred. Therefore, the SH-SAW biosensors had excellent stability and external interference could be excluded. The phase shift can be attributed to the mass added to the sensitive area, and thus, the authenticity of the data can be guaranteed. In addition, the endotoxin was pumped into the sensitive area without the aptamer being immobilized on it, which confirms that the phase shift was indeed caused by the specific binding of the aptamer and the endotoxin. The result is shown in Fig. 7b. The phase was kept in the steady state after the endotoxin was pumped into the reaction cell. Therefore, the phase shift can be guaranteed by the specific binding of the aptamer and the endotoxin. In addition, the phase shifts induced by pumping the PBS and aptamer into the sensitive area without the SLG film are shown in Fig. S4. The results revealed that the SLG film was used to immobilize the aptamer in the sensitive area by the crosslinking method.

**Fig. 8:**
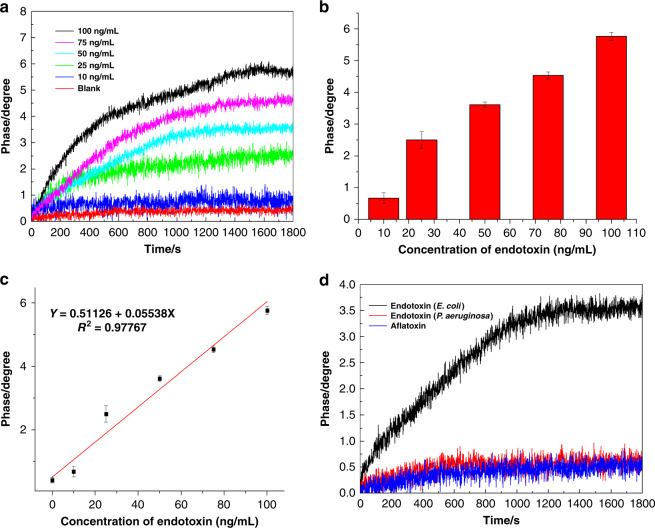
The sensitivity and selectivity of the SH-SAW biosensor with SLG film. **a** The real-time monitoring phase shift signals of the specific binding of the aptamer and the endotoxin at different concentrations. **b** Histogram of the specific binding of the aptamer to the endotoxin at different concentrations. **c** Standard curve for the quantitative detection of an endotoxin with an SH-SAW biosensor based on single-layered graphene. The phase shift on the *y*-axis had a linear relationship with the increasing concentrations of endotoxin on the *x*-axis. Each data point represents an average of at least three measurements ± the standard deviation. **d** The real-time phase signals of the endotoxin obtained from E. coli, the endotoxin obtained from P. aeruginosa, and the aflatoxin binding with the aptamer, which were all analyzed at concentrations of 50 ng/mL.

In the stable test environment, the performance of the SH-SAW biosensor with the SLG film was assessed by detecting the different concentrations of the endotoxin (blank, 10, 25, 50, 75, and 100 ng/mL). The real-time phase shifts are presented in Fig. 8a, and the corresponding histogram is shown in Fig. 8b. As one can see from the results, the phase shifts increased with increasing endotoxins concentration. The phase shifts were linear for the concentration of endotoxin in the range from blank to 100 ng/mL, with a correlation coefficient of 0.97767 (Fig. 8c). In the linear region, the calculated sensitivity was S ≈ 0.044 deg/ng/mL. The limit of detection was as low as 3.53 ng/mL, which was the blank concentration plus the three-fold standard deviation. The CVs of the ▵Phase obtained from different concentrations of endotoxin are shown in Table S3. The outcome of the experiment is also compared with some other reported results in Table 1.

**Table 1:** Comparison of the sensitivity and the LOD to those of other reported studies.

Method	Material	Detection	Linear range	LOD	Reference
Fluorescence quenching	Graphene oxide (GO)	Endotoxin	10–500 ng/mL	8.7 ng/mL	^46^
SH-SAW	Gold	Biotin	10–400 μg/mL	<6 μg/mL	^22^
SH-SAW	Gold	HIV	0.75–3 μg/ml	165 ng/mL	^23^
Field effect transistor	CVD graphene	ss-DNA	1 fM–1 pM	10 fM	^41^
Love-wave	Gold	Prostate antigen	10 ng/mL–1 μg/mL	10 ng/mL	^24^
SH-SAW	CVD single-layered graphene	Endotoxin	0–100 ng/mL	3.53 ng/mL	This work

The selectivity is especially important to the performance of an SH-SAW biosensor. A nontarget biological sample may cause a higher phase shift than the actual value, which would undoubtedly lead to incorrect experimental results. Therefore, the endotoxin obtained from P. aeruginosa and the aflatoxin were pumped into the reaction cell with a concentration of 50 ng/mL to test and verify the selectivity of the SH-SAW biosensor. As shown in Fig. 8d, the phase shifts of the endotoxin obtained from P. aeruginosa and the aflatoxin were almost the same as those of the blank solution, while the phase shift of the endotoxin with a concentration of 50 ng/mL was approximately 3.5°. The results reveal that the SH-SAW biosensor in this study had excellent selectivity in discriminating the endotoxin from the endotoxin obtained from P. aeruginosa and the aflatoxin.

## Conclusions

In this work, we reported a highly sensitive and label-free method for the detection of an endotoxin by an SH-SAW biosensor with SLG film. This technology proved the high sensitivity of the SH-SAW biosensor, and provided an effective platform for the detection of an endotoxin. The SH-SAW biosensor demonstrated a linear relationship with the concentration range of the endotoxin from 0 to 100 ng/mL, and a detection limit of 3.53 ng/mL was achieved. In addition, the stability and excellent specificity make the SH-SAW biosensor a promising alternative to conventional endotoxin detection methods. Therefore, an SH-SAW biosensor with SLG may offer a more effective and accurate prognosis evaluation in clinical diagnosis. However, the repeatability of this device was not ideal. In the future, this biosensor may be developed as a miniaturized and versatile device. However, more detailed work should be performed before clinical application.

## Supplementary information

An aptamer-based shear horizontal surface acoustic wave biosensor with a CVD-grown single-layered graphene film for high-sensitivity detection of a label-free endotoxin
