# Supplementary material for: An aptamer-based shear horizontal surface acoustic wave biosensor with a CVD-grown single-layered graphene film for high-sensitivity detection of a label-free endotoxin
Source: Microsyst Nanoeng. 2020 Feb 10;6:4. doi: 10.1038/s41378-019-0118-6 (PMC8433395; doi:10.1038/s41378-019-0118-6)
Supplement: Supplementary file 1 — An aptamer-based shear horizontal surface acoustic wave biosensor with a CVD-grown single-layered graphene film for high-sensitivity detection of a label-free endotoxin [file 41378_2019_118_MOESM1_ESM.pdf]

Supporting information

An aptamer-based shear horizontal surface acoustic wave biosensor with a CVD-grown single-layered graphene film for high-sensitivity detection of a label-free endotoxin

Junwang Ji[1], Yiquan Pang[2], Dongxiao Li[1], Zheng Huang[3], Zuwei Zhang[5], Ning Xue[4*], Yi Xu[1*], Xiaojing Mu[1*]

[1] Key Laboratory of Optoelectronic Technology & Systems, Ministry of Education, International R & D center of Micro-nano Systems and New Materials Technology, Chongqing University, Chongqing 400044, China.

[2] School of Chemistry and Chemical Engineering, Chongqing University, Chongqing 400030, China.

[3] Department of Applied Physics, Chongqing University, Chongqing 401331, China.

[4] Institute of Electronics, Chinese Academy of Sciences, Beijing 100190, China.

[5] Chongqing Acoustic-Optic-Electric Corporation, China Electronic Technology Group Corporation, Chongqing 400060, China

SI-1. MATERIALS AND METHODS

1.1. Fabrication of the Detection Cell with Micro-channels

The detection cell (Fig. S1) was designed using SolidWorks software and made of acrylic material, which consisted of inlet/outlet micro-channels. The whole detection cell is 7.5 centimeters long and 6 centimeters wide. The heights of the chip layer, flow channel layer, circuit layer are 1 centimeters, 0.5 centimeters, 1 centimeters respectively. The volume of the reaction cell, with a diameter of 4 mm and a height of 1.5 mm, was about 20 μL. To guarantee the non-leakage property of the sensitive area of the SH-SAW biosensor, a sealing ring made of silicon rubber (external diameter: 7 mm; inner diameter: 4 mm) and Nd-Fe-B magnets (diameter: 9 mm; height: 2 mm) were used. A spindle was used for ease of operation, and the liquid inlet/outlet was designed to introduce the tested solution. Four gold-tipped electrical spring probes were used to

contact the four pads of the SH-SAW biosensor through the spring probe holes so that the signal of the SH-SAW biosensor could be detected. Then these probes were welded onto the PCB boards and the SMA cables were used to connect the vertical network analyzer.

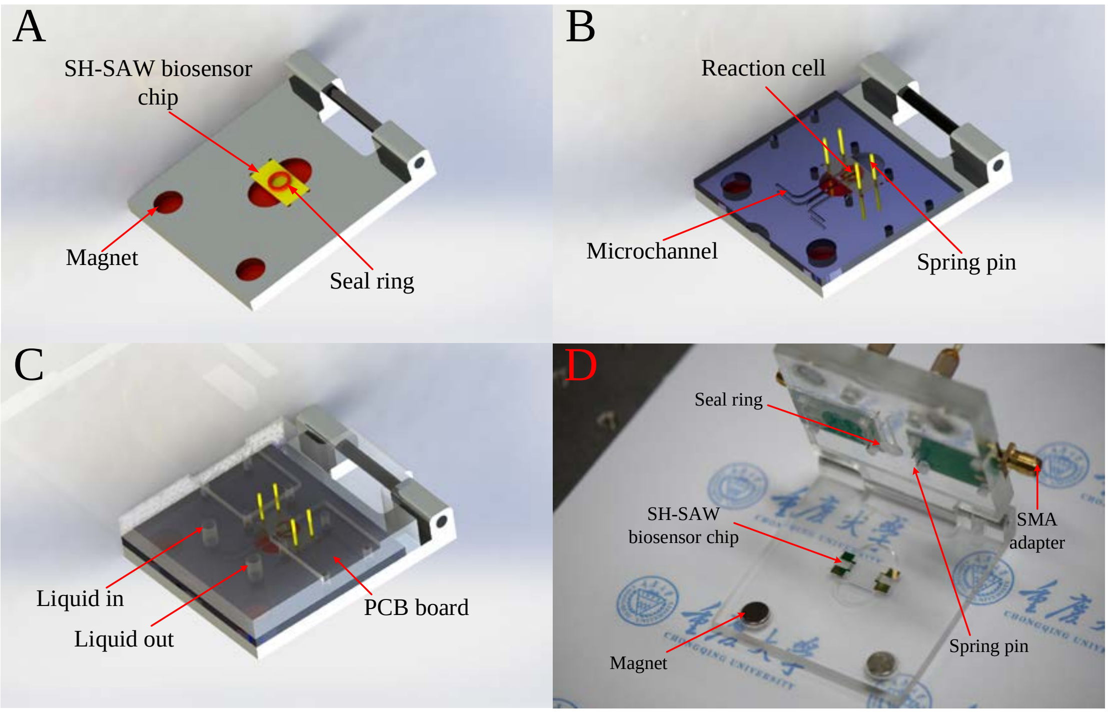

Fig S1. (A) Structure of the micro-channel detection cell: the chip layer, (B) the flow channel layer, (C) the circuit layer, (D) the real device

1.2. Electrical Measurements and Characterizations

The chemical compositions of graphene were characterized by X-ray photoelectron spectroscopy (XPS, EscaLab 250Xi, USA). To characterize the size of the IDTs and the surface topography of the SLG in the sensitive area, thermal field emission scanning electron microscope (TFESEM, JSM-7800M, Japan) was used. The Raman spectra of the SLG were collected using a Raman spectrometer (Renishaw plc, Invia Reflex, UK), and the Transmission Electron Microscope (TEM, JEM2100F, JEOL, Japan) was used to characterize the SLG sample. The surface topography of the sensitive area was examined by atomic force microscopy (AFM, Dimension EDGE, Brook, USA).

SI-2. RESULTS AND DISCUSSION

2.1. Characteristics of the SH-SAW Biosensor Chip

As shown in Fig. S2A, the base frequency was 246.2 MHz and the attenuation was 15.9 dB. The SH-SAW biosensor chip had 60 pairs of input IDTs and output IDTs (Table S1), and the width of the fingers was 5 μm, which signified that the wavelength was 20 μm (Fig. S2B, C).

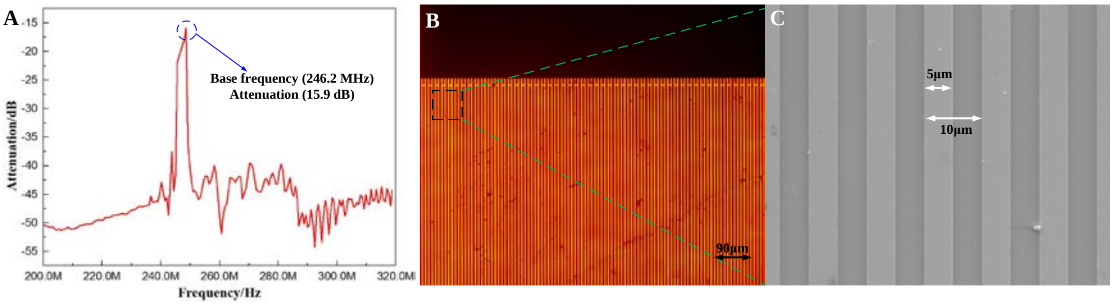

Fig. S2. (A) Spectrogram of the SH-SAW biosensor chip: the base frequency was 246.2 MHz and the attenuation was 15.9 dB. (B) Microscope image of the IDTs, (C) the SEM image of the IDTs indicates that the width of a single IDT was 5 μm and length of half cycle was 10 μm.

Table S1

Parameters of the SH-SAW biosensor chip

Parameters	Value	Unit
IDT finger width d=λ/4	5	μm
Sensitive area	6×3	mm
Thickness of the Cr/Au film	40/100	nm
Number of the input and output fingers	60×60	pair

To investigate the vibration and the SH wave characteristics, the 3D-FEA was performed using COMSOL 5.2a software. The model dimensions, geometry and boundary conditions used in 3D-FEA modelling is shown in Table S2. Input IDTs are defined by boundary conditions and arbitrary potential are applied (±10 V), they are modelled as massless conductors for calculation simplification. Applying this external electric field generates a strain proportional to the applied field. Additionally, The quartz material constants input into COMSOL are the elasticity (mechanical stiffness) matrix c_{ijkl}, the piezoelectric coupling matrix e_{ijk} and the permittivity matrix ε_{ij}.[1] The

resonant frequency $f_0 = v/ \lambda_0$, where v is The SAW mode propagating velocity and λ_0 is the acoustic wave length.[2] Low-reflecting boundary are used to absorb the wave at the boundaries of the outer surfaces, which will minimize reflections of elastic waves (pressure and shear waves) from these bounding surfaces. In this model x is taken to be the propagation direction, y is parallel to the SAW wavefront and z is normal to the surface. Fig S3 shows that the particle displacements were perpendicular to the SH-SAW propagation direction, which travelled along the substrate, and the vibration displacements parallel to the substrate along the Y direction. The propagation of the SH-SAW along the 36° X - 90° Y substrate was observed from the oblique view and front view.

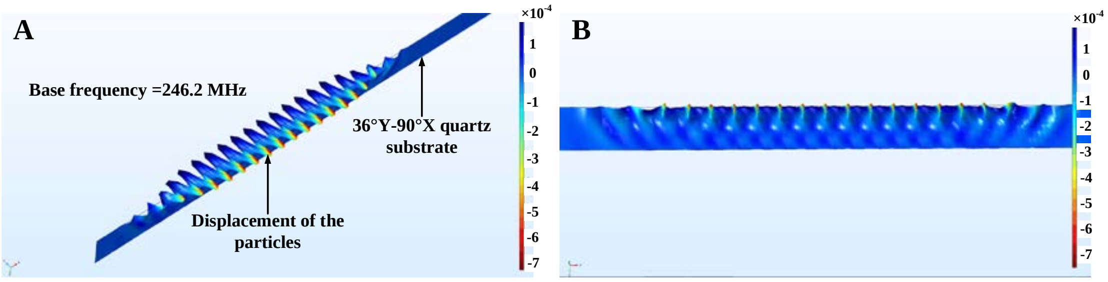

Fig S3. 3D simulated SH vibration displacement distribution along x direction, (A) oblique view of the surface of SH-SAW biosensor chip, (B) front view of the surface of SH-SAW biosensor chip.

Table S2

The model dimensions, geometry and boundary conditions used in 3D-FEA modelling.

Geometry	Value
Sensing length	152 μm
Sensing width	226 μm
Sensing height	18.5 μm
Pitch of electrodes	13.8 μm
Width of electrode	2.3 μm
Length of electrode	46 μm

Boundary	Condition
IDTs	Electric potential ±10V
Symmetric plane	Symmetry

Remaining boundaries	Zero charge

Table S3

Coefficients of variation of signals obtained from different endotoxin concentrations

The CVs of different endotoxin concentrations (%)					
0ng/mL	10ng/mL	25ng/mL	50ng/mL	75ng/mL	100ng/mL
19.4	24.1	10.2	2.3	2.3	2.3

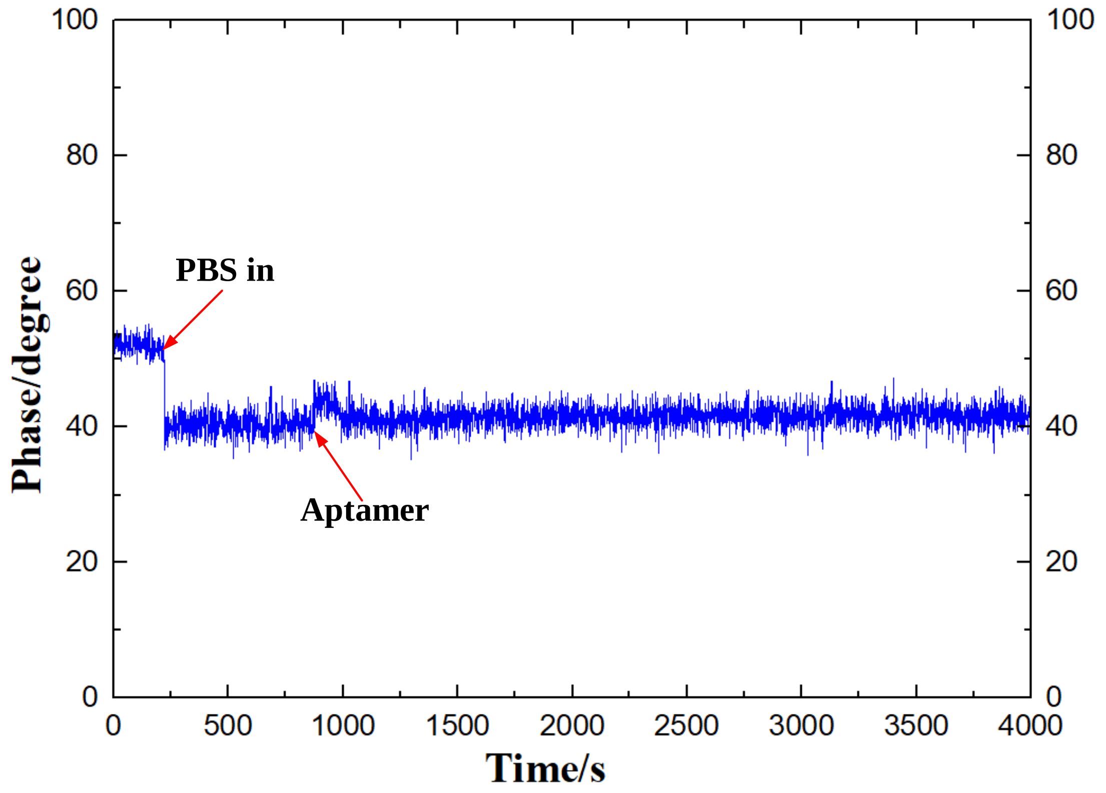

Fig S4. Phase shift induced by pumping the PBS and aptamer in the sensitive area without the SLG film in it.

REFERENCES

1 Brookes, J., Bufacchi, R., Kondoh, J., Duffy, D. M., McKendry, R. A., 2016. Determining biosensing modes in SH-SAW device using 3D finiteelement analysis. Sens. Actuators: B. 234, 412-419.

2 Ballantine, D. S., White, R. M., Martin, S.J., Ricco, A.J., Zellers, E.T., Frye, G.C., Wohltjen, H., 1997. Acoustic Wave Sensors, Theory, Design, and Physico-Chemical Applications. Academic Press.
